# Supplementary material for: Knowledge, attitudes and practices of community treatment supporters administering multidrug-resistant tuberculosis injections: A cross-sectional study in rural Eswatini
Source: PLoS One. 2022 Jul 14;17(7):e0271362. doi: 10.1371/journal.pone.0271362 (PMC9282659; doi:10.1371/journal.pone.0271362)
Supplement: S1 File — (DOCX) [file pone.0271362.s001.docx]

**S1: Knowledge, attitudes and practices of community treatment supporters questionnaire**

**FOR OFFICIAL PURPOSES ONLY**

Data Collector: ____________________

Date Collected: ____________________

Health Facility: ____________________

Please tick (✓) only one response that best answers the question. We would appreciate if you can answer all the questions as honestly as possible.

*Maka* (✓) *libhokisi lelicondzene nemphendvulo lekunetisako kulemibuto. Khetsa yinye imphendvulo ngaphandle nakushiwo kutsi tingaba tinengi. Yonkhe imibuto igcwaliswa ngekungativeti. Singajabula nawungayiphendvula yonkhe lemibuto ngekwetsembeka.*

**1. GENERAL AND DEMOGRAPHIC QUESTIONS**

1.1 How old are you?

*Unamingakhi iminyaka?*

| Under 30 years  *Phansi kwemashumi lamatsatfu* |  | 31-40 years  *Iminyaka leku 31-40* |  | 41-50 years  I*minyaka leku 41-50* |  | Over 50 years  *Ngetulu kweminyaka lelishumi nesihlanu* |  |
| --- | --- | --- | --- | --- | --- | --- | --- |

1.2 What is your gender?

*Ubulili buni?*

| *Wesilisa*/Male |  | Wesifazane/Female |  |
| --- | --- | --- | --- |

1.3 What is the highest level of education you have completed?

*Esikolweni wagcina kabani?*

| *Angifundzanga* No school |  | Primary school |  | High school |  | *Unyuvesi/ekilishi*  College/university |  | *Fundze kasebenta*  Literacy classes only |  |
| --- | --- | --- | --- | --- | --- | --- | --- | --- | --- |

1.4 *Uyasebenta yini nyalo?*

Are you employed?

| *Yebo*/Yes |  | *Cha*/No |  |
| --- | --- | --- | --- |

If yes please specify…………………..

1.5 Please answer the following regarding caregiver costs

*Tindleko talonaka logulako*

| No. | *Umuco* | Statement | Amount |
| --- | --- | --- | --- |
| 1.5.1 | *Umholo wenyanga* | Daily income |  |
| 1.5.2 | *Tindleko tekwekuhamba uma yehluke naleyo yalogulako* | Transport cost if different from patient |  |
| 1.5.3 | *Inombolo yemalanga ekungaveli emsebentini kwenteela kupheketela logulako* | No. of days lost from work to accompany patient |  |
| 1.5.4 | *Imali yemholo lephelele leyilahleke ngelilanga* | Amount of wages lost per day |  |

1.5 How many months have you been administering MDR-TB injections?

*Tingakhi tinyanga solo ujovisa umjovo we MDR-TB?*

|  | *Tinyanga*/months |
| --- | --- |

1.6 In the past 12 months, have you attended training on MDR-TB?

*Etinyangeni letilishumi nakuphili ukewaya kuyo fundze ngeMDR-TB?*

| Yebo/Yes |  | Go to question 1.6.1  Phendvula umbuto 1.6.1 |
| --- | --- | --- |
| Cha/No |  | Go to question 1.6.2 |

1.6.1 If you have attended training on MDR-TB, please indicate the topics that were covered:

*Nangabe uke wangenela tifundziso taka MDR-TB, cela ukhetse letihloko letafundziswa:*

|  | *Kuvikela kutselelwana kweMDR-TB* | MDR-TB infection control |
| --- | --- | --- |
|  | *Kulashwa kwe MDR-TB* | General MDR-TB management |
|  |  | MDR-TB injection administration |
|  | *Lokunye (chaza) …………………………………………………….………………………………………..* | Other (specify) ……………………………………………………………………………………………………….. ………........... |

1.6.2 If you have not attended training on MDR-TB in the past 12 months, please indicate why:

*Nawungakaze wayonfundza nge MDR-TB etinyangeni letilishumi nakubili letengcile, shano kutsi leni:*

|  | *Kufundza ngeMDR-TB akusebenti* | Training on MDR-TB is not useful |
| --- | --- | --- |
|  | *Kwakute lohlelo lokufundzisa nge MDR-TB lolwaluhleliwe* | No MDR-TB trainings were organised |
|  | *Bekute sikhatsi lesanele sekufundziswa iMDR-TB* | There was not enough time to attend MDR-TB training |
|  | *Bengite imali leyenele yekuyofundza ngeMDR-TB* | You could not afford the cost of attending MDR-TB training |
|  | *Lokunye (chaza) …………………………………………………………………................................................* | Other (specify) ……………………………………………………………………………………………………………… |

**2. LWATI NGE MDR-TB/ MDR-TB KNOWLEDGE**

*Ngekubuka lokwatiko nge MDR-TB, khombisa kutsi lemisho ikahle: Yebo(Y) Anginasiciniseko (U) Cha (N)*

Based on what you know about MDR-TB, please indicate whether the following statements are correct: Yes (Y); Unsure (U); and No (N).

| **No.** | ***Umuco*** | **Statement** | **Y** | **U** | **N** |
| --- | --- | --- | --- | --- | --- |
| 2.1 | *MDR -TB legciwane lengilapheki nge liphilisi i isoniazid ne rifampicin* | MDR-TB are strains of TB resistant to at least isoniazid and rifampicin |  |  |  |
| 2.2 | *MDR -TB itfolakala emoyeni* | MDR-TB is contagious |  |  |  |
| 2.3 | *Umgcugcuteli lonaka umuntfu lone MDR-TB angabanayo iMDR -TB* | A CTS providing care to a patient with MDR-TB may develop MDR-TB |  |  |  |
| 2.4 | *Bantfu labalala ekamelweni linye abasibo labangatselelana iTB* | People who sleep in the same room are not close TB contacts |  |  |  |
| 2.5 | *Bantfwana labangaphansi kweminyaka lemibili angeke babatselele batali babo noma labo lababanakako iMDR-TB* | Babies under two years are close TB contacts of their parents, or anyone who looks after them |  |  |  |
| 2.6 | *Umuntfu angayitfola iMDR-TB ngekuchawula lone MDR-TB* | A person can get MDR-TB from shaking hands with someone with MDR-TB |  |  |  |
| 2.7 | *Umuntfu lone HIV usematfubeni lamakhulu ekuba ne MDR-TB* | A person with HIV is more likely to develop MDR-TB |  |  |  |
| 2.8 | *Kuvula emafasitelo kungasita kuvikela kwandzisa iMDR-TB* | Opening windows can help in preventing the spread of MDR-TB |  |  |  |
| 2.9 | *Kufaka kwekuvikela emakhala iN95 kuyawehlisa ematfuba ekutfula iMDR-TB* | Wearing a N95 respirator can reduce the risk of transmission of MDR-TB |  |  |  |
| 2.10 | *Bonkhe labane MDR -TB banetimphawu letibonakalako.* | All people with MDR-TB infection have visible symptoms |  |  |  |
| 2.11 | *Kukwehlela ngulona phawu lolujwayelekile le MDR-TB.* | Coughing is the most common symptom of MDR-TB |  |  |  |
| 2.12 | *MDR -TB itfolakala kahle esitfombeni sasegesini* | MDR-TB is best diagnosed from a chest X-ray |  |  |  |
| 2.13 | *Indlela lekahle yekubona kuphumelela ekulashweni kwe MDR-TB kucwaninga sikhwehlela nekubuka kusebenta kwemaphalisi* | The correct way of assessing MDR-TB treatment outcome is through sputum culture and drug sensitivity testing (DST) |  |  |  |
| 2.14 | *MDR-TB iyalapheka* | MDR-TB can be cured |  |  |  |
| 2.15 | *Emaphilisi emjovo latfolakala emitfolaphilo angayelapha iMDR-TB* | General antibiotics given at the health centre can cure MDR-TB |  |  |  |
| 2.16 | *IMDR-TB ilapheka kahle nganawa emaphilisi: irifampicin, amikacin and levofloxacin only* | MDR-TB is best treated with following drug combination: rifampicin, amikacin and levofloxacin only |  |  |  |
| 2.17 | *Sikhatsi lesanele sekujovela iMDR-TB tinyanga letisiphohlongo* | The standard length of injection treatment for a newly diagnosed case of MDR-TB is 8 months |  |  |  |
| 2.18 | *Umjovo i Amikacin nguwona usetjentiswa nawusacal kujovela iMDR TB* | Amikacin is the drug that is used for injection during the intensive phase |  |  |  |
| 2.19 | *Sikhatsi sekulashelwa iMDR -TB singaba tinyanga letilishumi nesiphohlongo kuya kuletingemashumi lamabili nakune.* | The duration of treatment for MDR-TB is between 18 to 24 months |  |  |  |
| 2.20 | *Kulesinye sikhatsi bantfu labalashelwa iMDR - TB ababincono ngoba abawanatsi emaphilisi abo* | Sometimes people with MDR-TB do not get better because they do not take their medication |  |  |  |
| 2.21 | *Imitsi lenetikhomba tekufa noma kungcola lekutsite kufanelwe ilahlwe* | Medications with visible contamination or breaches of integrity (e.g. cracks, leaks) should be discarded |  |  |  |
| 2.22 | *Kushikisha ungakajovi kuyabehlisa buhlungu nawujova* | Swabbing before injections will minimize the pain during injection |  |  |  |
| 2.23 | *Kuvalwa kwetinyalitsi nawucedza kujova kungabanga kuhlatjwa tinyalitsi* | Recapping of used needles can cause needle-stick injuries |  |  |  |
| 2.24 | *Kunatsa emaphilisi ekuvikela iHIV (PEP) kungawehlisa ematfuba ekutfola ligciwane le HIV nabahlatjwe yinyalitsi labasebenta esibhedlela* | Taking antiretroviral drugs as post-exposure prophylaxis (PEP) can reduce the rate of infection in health care workers exposed to HIV through needle-stick injuries |  |  |  |
| 2.25 | *Ematfumba labekujovwa khona kufanelwe abikwe kunurse losebenta emmangweni lolapha iMDR-TB* | An infection or boil on the injection site is a side effect related to the injection that should be reported to the community MDR-TB nurse |  |  |  |

**3. MDR-TB ATTITUDES**

Please indicate how much you agree or disagree with each of the following statements. Strongly agree (SA); agree (A); unsure (U); disagree (D); or strongly disagree (SD).

*Khetsa kutsi uvumelana nalemicu kangakanani. Kakhulu kakhulu (SA) Kakhulu (A) Angati kahle (U) Ngiyaphikisa (D) Ngiphikisa Kakhulu (SD)*

| **No.** | ***Umuco*** | **Statement** | **SA** | **A** | **U** | **D** | **SD** |
| --- | --- | --- | --- | --- | --- | --- | --- |
| 3.1 | *MDR-TB iyinkinga kutemimango kaNgwane* | MDR-TB a major public health threat in Swaziland |  |  |  |  |  |
| 3.2 | *Ngiva ngatsi timfundziso teMDR TB emmangweni tanele* | I feel awareness of MDR-TB in my community is adequate |  |  |  |  |  |
| 3.3 | *Kufundziswa kwalabalashelwa iMDR-TB nebantfu bommango kuze kuvikeleke kwandza kwe MDR-TB* | Community awareness about MDR-TB is important in the control of the disease |  |  |  |  |  |
| 3.4 | *Ngiyacondza bumcoka bekungenela timfundziso tekuvikela iTB ngalokwejwayelekile* | I understand the importance of attending regular training on TB prevention |  |  |  |  |  |
| 3.5 | *Nginelwati lolwanele ngekulashwa kwe MDR-TB emmnagweni* | I have enough information about community MDR-TB management |  |  |  |  |  |
| 3.6 | *Ngumsebenti wami kufindzisa tigulane nge luvikelwa kweMDR-TB* | It is my responsibility to teach patients about TB prevention |  |  |  |  |  |
| 3.7 | *Bantfu labalashelwa iMDR-TB kununa bahlukaniswe kulabaneHIV* | Patients with known MDR-TB should be separated from HIV patients |  |  |  |  |  |
| 3.8 | *Kugeza tandla ngingakatsintsi sigulane noma sengicedzile kumcoka emebentini wami* | Washing my hands before and after direct patient contact is a necessary part of my work |  |  |  |  |  |
| 3.9 | *Ngiyakhutsata kuvulwa kwemafasitelo nekungenisa lilanga emakhaya ngaphandle kwekubuka simo selitulu* | I encourage adequate ventilation and sunlight at the patient home, regardless of weather conditions |  |  |  |  |  |
| 3.10 | *Ngiyayisebentisa imaskhi noma kungemnandzi* | I use a N95 respirator even though it may be uncomfortable |  |  |  |  |  |
| 3.11 | *Ngiyakhatsateka ngekutfola iMDR-TB ngisasebenta* | I worry about acquiring active MDR-TB disease while at work |  |  |  |  |  |
| 3.12 | *Ngicabanga ngisematfubeni lamacane ekutfola iMDR-TB kulenginakekelako* | I think I have a very low risk of acquiring MDR-TB from my patient |  |  |  |  |  |
| 3.13 | *Ngiyakholelwa kutsi kusebentisa kahle kwemijovo kungehlisa ematfuba ekutfola kugula kulabasebenta ngetemphilo* | I believe following safe injection practices can help reduce the risk of infectious adverse events in healthcare providers |  |  |  |  |  |
| 3.14 | *Ngicabanga kutsi kulukhuni kulabalashelwa iMDR-Tb kutsi bacondze kutsikufuna bachubeke nekunatsa emaphilisi kute babe ncono* | I think it is difficult for patients with MDR-TB to understand the need to continue taking medication after they start feeling better |  |  |  |  |  |
| 3.15 | *Ngingasho kutsi kungacedzi noma kungatsi kahle emaphilisi eMDR-TB angenta timphawo tibe nguletinkhulu kakhulu* | I consider interrupted MDR-TB treatment course to be a possible cause of worsening of symptoms |  |  |  |  |  |
| 3.16 | *Ngiyakholelwa kutsi kunatsa imitsi yesintfu noma lokunye kwenta kulashwa kweMDR – TB kubelukhuni* | I believe taking traditional or alternative medicine makes the treatment of MDR-TB difficult |  |  |  |  |  |
| 3.17 | *Ngiyeva kufuna ngibeneluvelo kulengimelaphako lolashelwa iMDR-TB* | I feel I should show compassion to my MDR-TB patient |  |  |  |  |  |
| 3.18 | *Labalashelwa iTB bangasoleka bona ngesimo sabo* | MDR-TB patients are to blame for their own condition |  |  |  |  |  |
| 3.19 | *Ngiyacabanga labalashelwa iMDR-TB bayagcekwa kakhulu emimangweni ngale sifo* | I feel MDR-TB patients are confronted with significant social stigma surrounding the disease |  |  |  |  |  |
| 3.20 | *Lengimsitako lolashelwa iMDR-TB angete afuna kutsu bantfu bati kutsi ine TB* | My MDR-TB patient may not want other people to know that they have TB |  |  |  |  |  |
| 3.21 | *3.14 Longiphetse usheshe atfolakale nangidzinga lusito ekunakeni lolashelwa iMDR-TB* | My supervisor is easily accessible when I need help in managing my MDR-TB patient |  |  |  |  |  |

**4. *LOKWENTIWA KA MDR-TB*/MDR-TB PRACTICES**

4.1.1 Do you have a CTS MDR-TB training manual?

*Unayo incwandzi yeMDR -TB yekufundzisa bagcugcuteli?*

| Yes  *Yebo* |  | Go to question 4.1.2  *Phendvula umbuto 4.1.2* |
| --- | --- | --- |
| No/*Cha* |  | Go to question 4.1.4  *Phendvula umbuto 4.1.4* |

4.1.2 How often do you refer to the CTS MDR-TB training manual?

*Uyisebentisa kangakanani lencwandzi yeMDR yekufundzisa bagcugcuteli.*

| Always  *Sonkhe sikhatsi* |  | Frequently  *Ngivamisile* |  | Rarely  *Ngazo* |  | Never  *Cha* |  |
| --- | --- | --- | --- | --- | --- | --- | --- |

4.2.1 Are you personally involved in educating patients or communities about MDR-TB?

*Ngekwakho uyabafundzisa labalashelwa iTB noma emmangweni ngeMDR-TB?*

| Yes/Yebo |  | No/Cha |  |
| --- | --- | --- | --- |

4.2.2 How often do you provide information on MDR-TB?

*Ulunika kanjani lwati ngeMDR-TB?*

| Always  *Sonkhe sikhatsi* |  | Frequently  *Njalo* |  | Rarely  *Ngazo* |  | Never  *Cha* |  |
| --- | --- | --- | --- | --- | --- | --- | --- |

4.3.1 How often is cross ventilation implemented in the room your MDR-TB patient sleeps?

*Ngabe kungeniswa kwemoya lokwanele kuyentiwa egumbini lapho kulala lomlaphela iMDR-TB?*

| Yes  Yebo |  | No  Cha |  |
| --- | --- | --- | --- |

4.3.2 If no, have you raised your concerns about this with the community MDR-TB team?

*Nangabe cha, uke wakuveta kulelicembu le MDR-TB emmangweni?*

| Yes  Yebo |  | No  Cha |  |
| --- | --- | --- | --- |

4.4.1 How often do you wear a N95 disposable respirator when attending to an MDR-TB patient?

*Uyifaka imaskhi iN95 nawuya esigulaneni sakho seMDR-TB?*

| Every time I am attending to an MDR-TB patient  *Sonkhe sikhatsi nanginaka lolashwa iMDR-TB?* |  | Frequently *Njalo* |  | Rarely  *Ngazo* |  | Never  *Cha* |  |
| --- | --- | --- | --- | --- | --- | --- | --- |

4.5.1 Are there enough supplies such as soap and clean water to wash your hands at patient homes?

*Ngabe emanti netinsipho enele kugeza tandla emakhaya alabalashwako?*

| Yes  Yebo |  | No  Cha |  |
| --- | --- | --- | --- |

4.5.2 Do you wash your hands before and after direct contact with an MDR-TB patient?

*Uyatigeza tandla ungattsintsi nanawucedza kunaka lolashelwa iMDR-TB?*

| Yes  Yebo |  | No  Cha |  |
| --- | --- | --- | --- |

4.5.3 How often do you wash your hands before and after direct contact with an MDR-TB patient?

*Utigeza kangakanani tandla ungakatsintsi nanawucedza kubona lolashelwa iMDR-TB?*

| Every time I am attending to an MDR-TB patient |  | Frequently |  | Rarely |  | Never |  |
| --- | --- | --- | --- | --- | --- | --- | --- |

4.6.1 How often do you use a clean needle and syringe to draw up and administer medication?

*Uyisebentisa kangakanani inyalitsi lehlobile nemjovo kunika umjovo?*

| Every time I am attending to an MDR-TB patient |  | Frequently |  | Rarely |  | Never |  |
| --- | --- | --- | --- | --- | --- | --- | --- |

4.6.2 How often do you immediately place needles and syringes in a sharps disposal container after administering an injection?

*Imijovo lesebentile netinyalitsi utibeka emgcomeni lolahla tinyalitsi ngekushesha?*

| Every time I am attending to an MDR-TB patient  Sonkhe sikhatsi nangi naka lone MDR-TB |  | Frequently  Kaningi |  | Rarely  Angikavami |  | Never  Angikwenti |  |
| --- | --- | --- | --- | --- | --- | --- | --- |

4.7.1 Have you ever sustained a needle stick injury during your practise as a CTS?

Wake wahlatjwa yinyalitsi usasebenta njengemgcugcuteli?

| Yes | Yebo | No | Cha |
| --- | --- | --- | --- |

4.7.2 Did you report the needle stick injury?

Wakubika kuhlatjwa yinyalithi?

| Yes | Yebo | Go to question 4.7.3  Phendvula umbuto 4.7.3 |
| --- | --- | --- |
| No | cha | Go to question 4.7.4  Phendvula umbuto 4.7.4 |

4.7.3 Did you take on HIV PEP after sustaining the needle stick injury?

*Wawanatsa emaphilisi ekuvikela iHIV PEP ekubeni uhlatjwe yinyalisi.*

| Yes | Yebo |  |
| --- | --- | --- |
| No | Cha |  |

4.7.4 What was your reason for not reporting needle stick injury? Please tick all that apply.

*Kwaba yini sizatfu sakho sekungabiki kuhlatjwa yinyalitsi? Khetsa lovumelana nako.*

| *Bengingeke ngicitse sikhatsi.* | I could not spare the time |
| --- | --- |
| *Bekungangihluphi.* | I could not be bothered |
| *Bekunematfuba lamancane ekutfola iHIV.* | There was a very low risk of HIV transmission at the time |
| *Lebekalashelwa*  *iMDR-TB abete i HIV.* | The MDR-TB patient you cared for at the time was HIV negative |
| *Bengingati kufanele ngibike* | Did not know I had to |
| *Bengingati ngibike njani* | Did not know how to |
| *Bengesaba kutsi simo sengati lesikhomba kuba khina kweligciwane sitangilahlekisela umsebenti nje mgcugcuteli.* | I was afraid a positive HIV test result would affect my career as a CTS |
| *Lokunye (Chaza)……………………………………………………………………………………………………….* | Other (specify)……………………………………………………………………………………………………….. |

**5 LEVEL OF AWARENESS OF MDR-TB AND TASK-SHIFTING**

***LIZINGA LEKWATI NGE MDR-TB NEKUSHINTJWA KWEMISEBENTI KUSUKELA KULABO LABACECESHIWE***

5.1 In your opinion, is MDR-TB a major public health threat in Swaziland?

*Ngekubuka kwakho, i-MDR-TB iyingoti yini emphilweni yebantfu eSwatini?*

| Yes |  | Unsure |  | No |  |
| --- | --- | --- | --- | --- | --- |
| Yebo |  | Angati |  | Cha |  |

5.2 Do you think community members should play a role in MDR-TB care?

*Uma ucabanga, ummango unayo yini indzima lewungayidlala ekunakeni labo labane MDR-TB?*

| Yes |  | Unsure |  | No |  |
| --- | --- | --- | --- | --- | --- |
| *Yebo* |  | *Angati* |  | *Cha* |  |

5.3 Are you aware of any task-shifting of responsibilities from professional nurses to community health workers in relation to medical conditions other than MDR-TB in Swaziland?

*Ingabe unalo lwati ngekushintjwa kwemisebenti kusuka kubahlengikati labaceceshiwe kuye etisebentini temphilo temmango macondzana naletinye timo temphilo ngaphandle kwe MDR-TB eSwatini?*

| Yes |  | Unsure |  | No |  |
| --- | --- | --- | --- | --- | --- |
| *Yebo* |  | *Angati* |  | *Cha* |  |

If yes, please give examples

*Uma utsite yebo, sicela tibonelo*

………………………………………………………………………………………………………………………………………………………………………………………………………………………………………………………………………………………………………………………………………………………………………

**6 ROLES AND RESPONSIBILITIES OF COMMUNITY TREATMENT SUPPORTERS (CTSs)**

***IMISEBENTI NETINDZIMA TEBASEKELI BEKWELAPHA BEMMANGO (EMA CTS)***

6.1 In your opinion, what type of CTS do you think is preferable?

*Ngekubuka kwakho, nguyiphi inhlobo yemsekeli wekwelapha ummango lokunguyena uncono?*

|  | *Umsekeli wekwelapha loceceshwe ngemijovo yeMDR-TB neDOT* |  | A specialist CTS (mainly for MDR-TB injections and DOT) |
| --- | --- | --- | --- |
|  | *Umsekeli loceceshwe ngetimo temphilo letehlukile njengeHIV/DOT/nalokunye lokuphatselene netemphilo emmangweni* |  | A generalist CTS (DOT/HIV issues/other health promotion roles in the community) |

Please explain

*Sicela uchaze*

………………………………………………………………………………………………………………………………………………………………………………………………………………………………………………………………………………………………………………………………………………………………………

**7 RISKS/BENEFITS OF USING CTSs IN MDR-TB INJECTION ADMINISTRATION**

***TINGOTI/NETINZUZO TEKUSEBENTISA BASEKELI BEKWELAPHA BEMMANGO EKUJOVELENI i-MDR-TB***

7.1 In your opinion, what are the three greatest risks related to using CTSs in administering MDR-TB injections, if any?

*Ngekubuka kwakho, yini tingoti letintsatfu letihambelana nekutsi basekeli bekwelapha ummango bajove ummango lone MDR-TB, uma ikhona?*

………………………………………………………………………………………………………………………………………………………………………………………………………………………………………………………………………………………………………………………………………………………………………………………………………………………………………………………………………………………………

7.2 In your opinion, what are the three greatest benefits related to using CTSs in administering MDR-TB injections, if any?

*Ngekubuka kwakho, yini tinzuzo letintsatfu letihambelana nekutsi basekeli bekwelapha ummango bajove ummango lone MDR-TB, uma ikhona?*

………………………………………………………………………………………………………………………………………………………………………………………………………………………………………………………………………………………………………………………………………………………………………………………………………………………………………………………………………………………………

**8 INCENTIVES AND COMPENSATION**

***IMBHADALO NEKUHOLA***

8.1 In your opinion, do you think CTSs should be compensated for the tasks they perform in MDR-TB care?

*Ngekubuka kwakho, basekeli bekwelapha ummango kumele bayitfole yini imbhadalo noma liholo ngemsebenti labawentako ekunakeni ummango macondzana ne MDR-TB?*

| Yes |  | Unsure |  | No |  |
| --- | --- | --- | --- | --- | --- |
| Yebo |  | Angati |  | Cha |  |

Please explain your choice

*Sicela uchaze* ………………………………………………………………………………………………………………………………………………………………………………………………………………………………………………………………………………………………………………………………………………………………………………………………………………………………………………………………………………………………

8.2 In your opinion, how should CTSs be compensated for the tasks they perform in MDR-TB care?

*Ngekubuka kwakho, basekeli bekwelapha ummango kumele babhadalwe kanjani ngalomsebenti labawentako ekunakeni ummango macondzana ne MDR-TB?*

………………………………………………………………………………………………………………………………………………………………………………………………………………………………………………………………………………………………………………………………………………………………………………………………………………………………………………………………………………………………

**9 RETENTION OF CTSs**

***KUGCINWA KWEBASEKELI BEKWELAPHA BEMMANGO***

9.1 What do you think can be done to retain CTSs in MDR-TB care?

*Ucabanga kutsi yini lengentiwa kugcina basekeli bekwelapha ummango macondzana ne MDR-TB?*

………………………………………………………………………………………………………………………………………………………………………………………………………………………………………………………………………………………………………………………………………………………………………………………………………………………………………………………………………………………………………………………………………………………………………………………………………………………

**10 POLICY REGULATION FOR TASK-SHIFTING**

***KULAWULWA KWEMIGOMO YEKUSHINTJWA KWEMISEBENTI KUSUKELA KULABO LABACECESHIWE***

10.1 In your view, should task-shifting of professional nurses’ responsibilities to CTSs in MDR-TB care be regulated?

*Ngekubuka kwakho, kushintjwa kwemisebenti kusukela kubahlengikati labaceceshiwe kuye kubasekeli bekwelapha ummango ekunakeni iMDR-TB kwamele kube nemigomo yini?*

| Yes |  | Unsure |  | No |  |
| --- | --- | --- | --- | --- | --- |
| Yebo |  | Angati |  | Cha |  |

Please explain your response

*Sicela uchaze*

………………………………………………………………………………………………………………………………………………………………………………………………………………………………………………………………………………………………………………………………………………………………………………………………………………………………………………………………………………………………

10.2 In your view, who should regulate the practice of CTSs in MDR-TB care?

*Ngekubuka kwakho, ngubani lekumele alawule imisebenti yebasekeli bekwelapha ummango ekunakeni i MDR-TB?*

………………………………………………………………………………………………………………………………………………………………………………………………………………………………………………

10.3 In your opinion, how can the practice of CTSs in MDR-TB care best be regulated?

*Ngekubuka kwakho, nguyiphi indlela lekahle yekwelawula imisebenti yebasekeli bekwelapha ummango labanake i MDR-TB?*

………………………………………………………………………………………………………………………………………………………………………………………………………………………………………………………………………………………………………………………………………………………………………………………………………………………………………………………………………………………………

**11 ACCEPTABILITY OF USING CTSs IN MDR-TB INJECTION ADMINISTRATION**

***KWEMUKELEKA KWEKUSEBENTISA BASEKELI NEKWELAPHA UMMANGO EKUJOVELENI I MDR-TB***

11.1 In your own opinion, should the task-shifting of MDR-TB injection administration to CTSs be adopted as one of a range of strategies to increase access to MDR-TB treatment?

*Ngekubuka kwakho, kushintjwa kwemsebenti kusuka kulabo labaceceshiwe kuye kubasekeli bekwelapha ummango ekujoveleni i MDR-TB kungaba yindlela yekukhulisa kutfololakala kwekwelapheka kwe MDR-TB?*

| Yes |  | Unsure |  | No |  |
| --- | --- | --- | --- | --- | --- |
| Yebo |  | Angati |  | Cha |  |

Please explain your response

*Sicela uchaze*

………………………………………………………………………………………………………………………………………………………………………………………………………………………………………………………………………………………………………………………………………………………………………………………………………………………………………………………………………………………………………………………………………………………………………………………………………………………

***Siyabonga sikhatsi sakho usagcwalisa lelifomu***

**Thank you for taking time to complete this questionnaire**
