# Supplementary material for: Knowledge, attitudes and practices of community treatment supporters administering multidrug-resistant tuberculosis injections: A cross-sectional study in rural Eswatini
Source: PLoS One. 2022 Jul 14;17(7):e0271362. doi: 10.1371/journal.pone.0271362 (PMC9282659; doi:10.1371/journal.pone.0271362)
Supplement: S2 File — (DOCX) [file pone.0271362.s002.docx]

**S2: Community treatment supporter observation checklist**

| **Checklist Item** | Yes | No |
| --- | --- | --- |
| **1 MDR-TB education and awareness** |  |  |
| 1.1 CTS MDR-TB training manual available |  |  |
| 1.2 Has patient disclosed MDR-TB status to his/her family? |  |  |
| 1.3 Have household members been screened for MDR-TB? |  |  |
| **2 DOT** |  |  |
| 2.1 Did the patient swallow the MDR-TB medicine in the presence of the CTS? |  |  |
| 2.2 From the patient card, how many times has the CTS missed giving injections and oral drugs |  |  |
| **3 Infection control** |  |  |
| 3.1 Does the patient sleep alone in a separate room? |  |  |
| 3.2 Does the room have windows? |  |  |
| 3.3 Windows in the patient’s room open |  |  |
| 3.4 CTS wearing N95 respirator |  |  |
| 3.5 Patient wearing surgical mask |  |  |
| 3.6 Is there adequate supply of soap and clean water to wash hands |  |  |
| **4 Safe injection handling technique** |  |  |
| 4.1 Hands washed before procedure |  |  |
| 4.2 New single needle and single syringe used |  |  |
| 4.3 Checks the vial for content, dose, and expiration date |  |  |
| 4.6 Fills syringe with contents of the vial |  |  |
| 4.7 Expels air from syringe |  |  |
| 4.8 Careful disposal of the drawing up needle from syringe and replace with a fresh one |  |  |
| 4.9 Locates the exact site for injection |  |  |
| 4.10 Disinfects the injection site with alcohol prep pad |  |  |
| 4.11 The patient is told to relax the muscle |  |  |
| 4.12 Inserts the needle swiftly at an angle of 90 degrees |  |  |
| 4.13 Aspirates briefly to ensure the needle is not sited in a blood vessel |  |  |
| 4.14 Injects all contents of the syringe slowly (less painful) |  |  |
| 4.15 Gently presses the injection site with a clean cotton ball |  |  |
| 4.16 Needle and syringe are disposed intact immediately in a puncture resistant sharps container |  |  |
| 4.17 Hands washed after procedure |  |  |
| 4.18 Records information on the patient’s card and other data collection forms |  |  |
