# Supplementary material for: Knowledge, attitudes and practices of community treatment supporters administering multidrug-resistant tuberculosis injections: A cross-sectional study in rural Eswatini
Source: PLoS One. 2022 Jul 14;17(7):e0271362. doi: 10.1371/journal.pone.0271362 (PMC9282659; doi:10.1371/journal.pone.0271362)
Supplement: Attachment — Submitted filename: Response to Reviewers.docx [file pone.0271362.s005.docx]

Centre for Development Support

Faculty of Economic and Management Sciences

University of the Free State

P.O. Box 399

Bloemfontein 9300

South Africa

Academic Editor

PLOS ONE

13 June 2022

**REF: Revision and resubmission of manuscript PONE-D-22-07174**

Dear Editor

Thank you for your email and the opportunity to revise our paper on ‘Knowledge, attitudes and practices of community treatment supporters administering multidrug-resistant tuberculosis injections: a cross-sectional study in rural Eswatini.’ The suggestions offered by the reviewer have been immensely helpful in improving some important aspects of the manuscript.

We have included the academic editor and reviewers’ comments (in italics) immediately after this letter and responded to them individually, indicating exactly how we addressed each comment and describing the changes we have made. The revisions have been approved by all authors. The changes in the revised manuscript accompanying this letter are marked up.

We hope the revised manuscript will better suit PLOS ONE, but will be happy to consider further revisions and we thank you for your continued interest in our research.

Sincerely,

Ernest Peresu

**Academic Editor and Reviewer Comments and Author Responses**

**Academic Editor**

***Comment 1:*** *Please ensure that your manuscript meets PLOS ONE's style requirements, including those for file naming.*

**Response:** Thank you for this reminder. We have revised our manuscript to ensure that it aligns with PLOS ONE's style requirements.

***Comment 2:*** *Your ethics statement should only appear in the Methods section of your manuscript. If your ethics statement is written in any section besides the Methods, please delete it from any other section.*

**Response:** We highly appreciate your comment. The ethics statement now only appears in the Methods section of our manuscript.

***Comment 3:*** *We note that you have indicated that data from this study are available upon request. In your revised cover letter, please address the following prompts:*

*a) If there are ethical or legal restrictions on sharing a de-identified data set, please explain them in detail (e.g., data contain potentially sensitive information, data are owned by a third-party organization, etc.) and who has imposed them (e.g., an ethics committee). Please also provide contact information for a data access committee, ethics committee, or other institutional body to which data requests may be sent.*

*b) If there are no restrictions, please upload the minimal anonymized data set necessary to replicate your study findings as either Supporting Information files or to a stable, public repository and provide us with the relevant URLs, DOIs, or accession numbers. For a list of acceptable repositories, please see* [*http://journals.plos.org/plosone/s/data-availability#loc-recommended-repositories*](http://journals.plos.org/plosone/s/data-availability#loc-recommended-repositories)*.*

**Response:** Thank you for reminding us about the importance of making data from this study available. We have uploaded the minimal anonymised data set as a Supporting Information file (S3).

***Comment 4:*** *Please review your reference list to ensure that it is complete and correct.*

**Response:** Thank you for this reminder and we have performed another thorough review of our reference list to ensure its both correct and complete.

**Reviewer 1**

***General comment:*** *This is a very interesting and relevant study. The findings and recommendations on appropriate task-shifting of basic health services to lay community members, in resource-limited settings will be helpful for further research.*

**Response:** We would like to thank Reviewer 1 for the thoughtful and thorough assessment of our manuscript. We are indeed grateful for the insightful comments.

***Comment 1:*** *Author should complete the omission in the sentence, "Instead of making trips to the . . .., MDR-TB patients receive their daily injections and DOT from CTSs in their (the patients’) homes."*

**Response:** Thank you for pointing this out. The revised sentence now reads: ‘Instead of making trips to the clinics, MDR-TB patients receive their daily injections and DOT from CTSs in their (the patients’) homes’ (Page 4)

**Reviewer 2**

***General comment:*** *The authors’ address a topical subject of relevance to the target audience of PLOS ONE using both quantitative methods to assess the knowledge, attitudes and practices of community treatment supporters administering multidrug-resistant tuberculosis injections in rural Eswatini. This should be encouraged.*

**Response:** We would like to thank the reviewer for carefully and thoroughly reading our manuscript and for the thoughtful comments and constructive suggestions. We are indeed grateful to the reviewer for his positive and encouraging comments.

***Comment 1:*** *In the methodology section, authors did not include the number patient homes where observations were conducted on the CTSs actual practice. Authors to include the number of CTSs that were sampled for observation in the methodology section of the abstract.*

**Response:** The methods section of the abstract has been improved by highlighting the number of CTSs that were sampled for direct observation (Page 2).

***Comment 2:*** *Authors to include description of MDR-TB drug regimen in use at the time of the study as part of the background.*

**Response:** We appreciate this comment and have accordingly added the description of MDR-TB drug regimen in use at the time of the study as part of the background (Page 4).

***Comment 3:***	*In the sampling sub-section of the methodology, the authors describe how the 20 CTSs were selected through a proportionate random sampling from the 3 main facilities after a stratified sampling. The distribution of the 82 purposively selected CTSs among the 3 facilities which form the sampling frame for the stratified sampling is unclear. Clearly describe the distribution of the 82 purposively selected* *CTSs among the 3 main facilities. This will clarify the size of the sampling frame.*

**Response:** We found this comment very helpful. We have rephrased and added more clarity on the distribution of the 82 CTSs among the 3 main facilities (Page 7).

***Comment 4:*** *In the Instrument development and measures sub-section, paragraph 2 (line 12) the authors describe Table 4 as having 12 questions assessing CTSs’ self-reported practices for MDR-TB. A review of table 4 shows responses to 11 questions rather than 12. Authors to review table 4 and update accordingly.*

**Response:** Thank you for pointing this out and we have reviewed the table accordingly. The sentence now reads: ‘CTS’s self-reported practices regarding MDR-TB were assessed based on responses to 11 questions as shown in Table 4’ (Page 9).
