# Supplementary material for: Knowledge, attitudes and practices of community treatment supporters administering multidrug-resistant tuberculosis injections: A cross-sectional study in rural Eswatini
Source: PLoS One. 2022 Jul 14;17(7):e0271362. doi: 10.1371/journal.pone.0271362 (PMC9282659; doi:10.1371/journal.pone.0271362)
Supplement: Attachment — Submitted filename: Reviewers comments PONE-D-22-07174_Updated.docx [file pone.0271362.s006.docx]

Reviewer Recommendation and Comments for UPDATED Manuscript Number PONE-D-22-07174

**Manuscript Number**: PONE-D-22-07174

The authors’ address a topical subject of relevance to the target audience of PLOS ONE using quantitative methods to assess the knowledge, attitudes and practices of community treatment supporters administering multidrug-resistant tuberculosis injections in rural Eswatini. This should be encouraged. The authors have made the required minor revisions to the manuscript (highlighted below) and the manuscript is recommended to the editor for publication.

1. **Abstract**

In the methodology section, authors did not include the number patient homes where observations were conducted on the CTSs actual practice.

**Action:** Authors to include the number of CTSs that were sampled for observation in the methodology section of the abstract.

**Comment:** This has been addressed by the authors

2. **Introduction**

This paper is a deep dive into practical aspects of MDR-TB management and goes to length to describe the KAP of the CTSs on administering MDR-TB injections. The field of DR-TB management is a rapidly changing field as treatment regimen and recommendations are changed as new evidence is available. There is no description by the authors of the treatment regimen including type of injectable that was used in the country by the CTSs. This is critical information to help readers underground the program context/background better.

**Action:** Authors to include description of MDR-TB drug regimen in use at the time of the study as part of the background.

**Comment:** This has been sufficiently addressed by the authors.

3. **Methods:**
a) In the ***sampling sub-section*** of the methodology, the authors describe how the 20 CTSs were selected through a proportionate random sampling from the 3 main facilities after a stratified sampling. The distribution of the 82 purposively selected CTSs among the 3 facilities which form the sampling frame for the stratified sampling is unclear.

### Action: Clearly describe the distribution of the 82 purposively selected CTSs among the 3 main facilities. This will clarify the size of the sampling frame

### Comment: This has been sufficiently described by the authors

b) In the ***Instrument development and measures sub-section***, paragraph 2 (line 12) the authors describe Table 4 as having 12 questions assessing CTSs’ self-reported practices for MDR-TB. A review of table 4 shows responses to 11 questions rather than 12.

**Action:** Authors to review table 4 and update accordingly.

**Comment:** This has been addressed by the authors

Thank you
